# A randomized, controlled trial of initial anti-retroviral therapy with abacavir/lamivudine/zidovudine twice-daily compared to atazanavir once-daily with lamivudine/zidovudine twice-daily in HIV-infected patients over 48 weeks (ESS100327, the ACTION Study)

**DOI:** 10.1186/1742-6405-6-3

**Published:** 2009-04-09

**Authors:** Princy N Kumar, Patricia Salvato, Anthony LaMarca, Edwin DeJesus, Parul Patel, Daniel McClernon, Allison Florance, Mark S Shaefer

**Affiliations:** 1 Georgetown University Medical Center, Washington, DC, USA; 2 Diversified Medical Practice, Houston, TX, USA; 3 Therafirst Medical Centers Inc, Ft. Lauderdale, FL, USA; 4 Orlando Immunology Center, Orlando, FL, USA; 5 Previous address: GlaxoSmithKline, Research Triangle Park, NC, USA

## Abstract

**Background:**

Traditional first line regimens containing a non-nucleoside reverse transcriptase inhibitor or protease inhibitor may not be suitable for a subset of antiretroviral-naïve patients such as those with certain co-morbidities, women of child-bearing potential, and intolerability to components of standard first line therapy. This study was conducted to determine if alternate treatment options may meet the needs of both general and special patient populations. The ACTION study was a randomized, open-label, multicenter, 48-week trial that compared the safety and efficacy of a triple nucleoside regimen versus a protease inhibitor plus a dual nucleoside regimen in HIV-1 treatment-naïve subjects.

**Results:**

279 HIV-infected subjects with HIV-1 RNA (VL) >5000 but < 200,000 copies/mL (c/mL) and CD4+ count ≥ 100 cells/mm^3 ^were randomized (1:1) to receive abacavir sulfate/lamivudine/zidovudine (ABC/3TC/ZDV) twice-daily or atazanavir (ATV) once-daily plus lamivudine/zidovudine (3TC/ZDV) twice-daily. Protocol-defined virologic failure was based on multiple failure criteria.

Non-inferiority of ABC/3TC/ZDV to ATV+3TC/ZDV was established with 62% vs. 59% of subjects achieving a VL < 50 c/mL at week 48, [ITT(E), M/S = F, 95% CI: -5.9, 10.4]. Similar results were observed in the 230 (82%) subjects with baseline VL<100,000 c/mL (ABC/3TC/ZDV vs. ATV+3TC/ZDV), 66% vs. 59%; 95% CI: -5.6, 19.5. However, ABC/3TC/ZDV did not meet the non-inferiority criterion compared to ATV+3TC/ZDV in the 48 subjects with baseline VL ≥ 100,000 c/mL, 39% vs. 60%; 95% CI: -49.2, 7.4, respectively. Protocol-defined virologic failure was similar between groups.

**Conclusion:**

ABC/3TC/ZDV demonstrated comparable virologic efficacy to ATV+3TC/ZDV in this population over 48 weeks. In those with a baseline VL ≥ 100,000 c/mL, subjects in the ATV+3TC/ZDV showed better virologic efficacy. Both regimens offer benefits in select therapy-naïve subjects.

**Trial Registration:**

[Clinical Trials Identifier, NCT00082394].

## Background

For the majority of ART-naïve, HIV-1 infected patients, the choice of an initial antiretroviral regimen continues to contain either a non-nucleoside reverse transcriptase inhibitor (NNRTI) or a boosted protease inhibitor (PI) in combination with two nucleoside reverse transcriptase inhibitors (NRTIs) [1]. However, there is an important subset of patients for which these first line regimens are unsuitable for a variety of reasons including presence of co-morbidities such as metabolic syndrome, underlying severe depression, drug-drug interactions, inability to tolerate low-dose ritonavir, and women of child-bearing potential. Additionally, as the population ages, patients are more likely to be on polypharmacy. For these groups of patients, it is important to select a regimen that is both individualized and provides suitable efficacy and safety compared to first-line regimens. The present study was conducted to determine if alternate treatment options may meet the needs of both general and special patient populations.

Triple NRTI therapy with the fixed dose combination of abacavir sulfate, lamivudine and zidovudine (ABC 300 mg, 3TC 150 mg, ZDV 300 mg; Trizivir^® ^GlaxoSmithKline, Research Triangle Park, NC) has several advantages to other HAART regimens including convenience to facilitate adherence, low pill burden, no food or water restrictions, generally favorable safety and metabolic profile, and few drug-drug interactions. The safety and efficacy of abacavir sulfate, lamivudine, and zidovudine (ABC/3TC/ZDV) or its components have been demonstrated in several randomized trials of ART-naïve subjects [2-4].

Study CNA3014 was an open-label, randomized, multicenter trial comparing triple nucleoside therapy with abacavir sulfate (ABC 300 mg; Ziagen^®^, GlaxoSmithKline, Research Triangle Park, NC) plus fixed dose lamivudine/zidovudine (3TC 150 mg, ZDV 300 mg; Combivir^®^, GlaxoSmithKline, Research Triangle Park, NC) both administered twice-daily (BID) versus indinavir (IDV) thrice-daily + 3TC/ZDV BID in ART-naïve subjects. At week 48, 60% of subjects in the ABC+3TC/ZDV group and 50% in the IDV+3TC/ZDV group achieved a plasma HIV-1 RNA (VL) <50 c/mL. In stratified analyses, results were similar among groups regardless of screening VL (< or ≥ 100,000 c/mL) in contrast to lower virologic responses observed with ABC+3TC/ZDV in subjects with VL >100,000 c/mL in an earlier double-blind, placebo-controlled study, CNAAB3005 [2,3].

Study ESS40002 was a randomized, open-label, three-arm, international, 96-week trial that evaluated 1) ABC/3TC/ZDV, 2) NFV+3TC/ZDV, and 3) NFV+3TC+stavudine (d4T) in a racially diverse ART-naive population of which 50% were female. Through 96 weeks, 41% vs. 39% vs. 33% of ABC/3TC/ZDV, NFV+3TC/ZDV, and NFV+3TC+d4T treated subjects, respectively, achieved a VL <50 c/mL. Subjects with a low baseline VL (<100,000 c/mL) responded comparably regardless of randomized treatment. In contrast, subjects in the ABC/3TC/ZDV group with a higher baseline VL (>100,000 but <200,000 c/mL) demonstrated greater virologic response (53%) compared with NFV+3TC/ZDV (29%) and NFV+3TC+d4T (42%) at week 96 [4].

The AIDS Clinical Trial Group (ACTG) 5095 study team found that triple nucleoside therapy with ABC/3TC/ZDV resulted in a significantly shorter time to virologic failure regardless of baseline viral load when compared to a pooled EFV-containing regimen of ABC/3TC/ZDV and 3TC/ZDV [5]. At week 48, 61% of subjects receiving ABC/3TC/ZDV compared with 83% on pooled EFV-containing regimens achieved an HIV-1 RNA <50 c/mL, the superior virologic response of the latter attributable to EFV. Inferior virologic responses among subjects randomized to the ABC/3TC/ZDV arm resulted in early termination of this study arm and removal of triple nucleoside regimens as a first-line therapy option from the US Department of Health and Human Services (DHHS) guidelines in ART-naïve subjects [6]. Surprisingly, this trend had not been observed in earlier trials with ABC/3TC/ZDV when compared to PI-based regimens with baseline VL up to 300,000 c/mL [2,3]. Therefore, given the totality of data on the safety, tolerability, and efficacy of ABC/3TC/ZDV compared to other initial ART regimens and consistent with the DHHS Guidelines, the combination of ABC/3TC/ZDV remains a reasonable option for the treatment of select ART-naïve subjects.

Atazanavir sulfate (ATV; Reyataz^®^, Bristol-Myers Squibb, Princeton, NJ) is a protease inhibitor that may also be a useful alternative therapy option in select patient populations. Atazanavir has several attributes including once-daily (QD) administration without ritonavir in ART-naïve subjects and a favorable lipid profile. However, scleral icterus and jaundice secondary to bilirubin elevations have been reported in subjects receiving atazanavir [7]. The safety and efficacy of atazanavir has been demonstrated in several randomized trials. Study AI424-034 was a pivotal, randomized, double blind, multicenter trial comparing atazanavir QD versus efavirenz QD each in combination with the 3TC/ZDV fixed-dose combination tablet BID. Through 48 weeks of therapy, 32% vs. 37% of subjects treated with ATV+3TC/ZDV vs. EFV+3TC/ZDV maintained viral suppression below 50 c/mL [8].

In a select population of ART-naïve HIV-1 infected subjects with low viral loads, ABC/3TC/ZDV and ATV+3TC/ZDV may offer similar benefits versus ritonavir-boosted PI or NNRTI-containing regimens. Therefore in the ACTION study, [Clinical Trials Identifier, NCT00082394], we compared the safety, tolerability, and efficacy of ABC/3TC/ZDV versus atazanavir plus 3TC/ZDV over 48 weeks in ART-naïve HIV-1 infected subjects with baseline VL ≥ 5000 and <200,000 c/mL.

## Methods

### Study Design

ESS100327 was a phase IV, randomized, non-inferiority, open-label, 48 week study comparing a fixed dose combination triple nucleoside regimen containing abacavir sulfate, lamivudine, and zidovudine (ABC/3TC/ZDV) administered twice-daily to a protease inhibitor based regimen of atazanavir (ATV) administered once daily with a fixed dose tablet of two nucleosides, lamivudine and zidovudine (3TC/ZDV), administered twice daily. Patients were recruited from 46 sites in the USA and Mexico and were eligible for enrollment if they were infected with HIV-1, aged 18 years or older, ART-naïve and had plasma HIV-1 RNA ≥ 5000 but <200,000 c/mL and CD4+ cell count ≥ 100 cells/mm^3^. Subjects were excluded if they had medical conditions or required medications that could compromise their safety or interfere with drug absorption, or if they had protocol-specific abnormal laboratory values.

This study was approved by ethics committees at each participating site and all subjects provided written informed consent. The study was conducted in accordance with Good Clinical Practice.

Treatment allocation was based on a central randomization schedule stratified by screening HIV-1 RNA (< or ≥ 100,000 c/mL). Patients were randomized 1:1 to receive either ABC/3TC/ZDV as one tablet twice daily (BID) or 2–200 mg capsules of ATV once daily (QD) plus one tablet of 3TC/ZDV administered BID. Subjects randomized to ABC/3TC/ZDV and diagnosed with a suspected abacavir hypersensitivity reaction (ABC HSR) were permitted a switch to 3TC/ZDV BID + tenofovir disoproxil fumarate (TDF) 300 mg QD. Subjects randomized to ATV+3TC/ZDV who experienced ATV treatment-limiting toxicity (specifically jaundice or scleral icterus secondary to bilirubin elevations) were permitted a switch to fosamprenavir calcium (FPV) 1400 mg BID + 3TC/ZDV BID.

### Assessments and Outcomes

Patients were evaluated at screening, Day 1 (baseline), and at weeks 2, 4, 8, 12, 16, 20, 24, 32, 40, and 48 and withdrawal. Clinical and laboratory assessments including HIV-1 viral load, CD4+/CD8+ lymphocyte subsets, clinical chemistry, hematology, serum lipid panels, insulin, and hemoglobin A1C were performed regularly throughout the study. Insulin resistance and sensitivity were assessed on fasting subjects periodically using the homeostasis model assessment of insulin resistance (HOMA-IR) and the quantitative insulin-sensitivity check index (QUICKI), respectively. US Centers for Disease Control and Prevention (CDC) classification was assessed and samples for hepatitis B and C serology and pregnancy tests were performed at baseline and at investigator discretion, where appropriate. Plasma for genotypic drug resistance testing was collected at baseline and at any confirmatory visits for virologic failure.

HIV-1 RNA was measured by the Roche COBAS Amplicor HIV-1 Monitor test, Version 1.5 and the Roche COBAS Amplicor HIV-1 Ultrasensitive Monitor test, Version 1.5 (Branchburg, NJ, USA). Genotypic analyses were performed by GlaxoSmithKline Clinical Virology (Research Triangle Park, NC) using the TruGene HIV-1 Genotyping assay and by Monogram Biosciences (South San Francisco, CA, USA) using the Phenosense HIV assay, respectively. All other laboratory testing was performed centrally by Quest Laboratories (Van Nuys, CA, USA).

The primary efficacy endpoint was the proportion of subjects with HIV-1 RNA <50 c/mL at week 48 who did not meet the definition of virologic failure through week 48 using an intent-to-treat exposed analysis [ITT(E)]. Due to the concerns raised by the ACTG 5095 study, multiple, stringent criteria for protocol-defined virologic failure was established as shown in Table 1. The primary safety endpoints were the frequency of treatment-limiting adverse events, Grade 2–4 adverse events, and serious adverse events over 24 and 48 weeks.

**Table 1:** Virologic Failure Definition

**Prior to or at Week 24**
1. Failure to have ≥ 1 log HIV-1 RNA drop from baseline by week 12
2. Reduction of plasma HIV-1 RNA to <50 copies/mL on two occasions followed by increase of ≥ 400 copies/mL on two consecutive times prior to week 24*
3. Failure to have <400 copies/mL by week 24

**After Week 24**

4. Plasma HIV-1 RNA ≥ 400 copies/mL on two consecutive occasions after week 24*
5. Subject had an HIV-1 RNA ≥ 400 copies/mL at week 48 with confirmation

* virologic rebound any time at or after confirmed virologic failure of ≥ 1265 copies/mL on two consecutive occasions resulted in subject's discontinuation from the study as a virologic withdrawal.

Secondary endpoints included the proportion of subjects with HIV-1 RNA <50 c/mL at weeks 24 and 48 in those not meeting protocol-defined virologic failure. Subjects with regimen switches for suspected ABC HSR or ATV treatment-limiting toxicity were analyzed as treatment failures (switch = failure, S = F) and in sensitivity analyses in which the switch was not treated as a failure. Additional secondary endpoints included proportion of subjects with HIV-1 RNA <400 c/mL at weeks 24 and 48, change in plasma HIV-1 RNA and CD4+ cell count from baseline, time to loss of virologic response (TLOVR), change in fasting lipids, insulin, and glucose, adherence, and development of genotypic and phenotypic resistance at virologic failure.

### Statistical Analysis

Efficacy and safety analyses included all subjects that were treated before withdrawal (intent-to-treat exposed [ITT(E)]). Assuming a success rate of 0.58 for the ABC/3TC/ZDV BID group and 0.50 for the ATV QD + 3TC/ZDV BID group, a total of 280 (140 subjects per treatment arm) provided greater than 90% power (one-sided, α = 0.025) to establish non-inferiority of ABC/3TC/ZDV compared to ATV+3TC/ZDV using a 12% non-inferiority margin. Non-inferiority was established if the lower limit of the two-sided 95% confidence interval for the difference in proportions (ABC/3TC/ZDV BID – ATV QD + 3TC/ZDV BID) exceeded -12%.

The primary analysis was ITT(E) where missing or regimen switches for ABC HSR or ATV treatment-limiting toxicity were treated as failures (M/S = F). Additional analyses included ITT(E), M = F where the regimen switches were not treated as failures. Observed analyses in which missing data was not imputed were also performed with and without the regimen switches treated as failures. For the primary efficacy analysis and the corresponding sensitivity analyses, the treatment response rates in each group were stratified by the baseline HIV-1 RNA (< or ≥ 100,000 c/mL) using Mantel-Haenszel weights. Exploratory analyses in each baseline viral load stratum were also performed.

Descriptive statistics were used to evaluate the secondary efficacy endpoints. ITT(E) and Observed analyses were carried out for the proportions of subjects with plasma HIV-1 RNA < 400 c/mL and < 50 c/mL at week 48 using M/S = F and M = F, overall and stratified by baseline viral load stratum. Kaplan-Meier estimates were calculated for the time to first study-defined virologic failure and the time to loss of virologic response (TLOVR).

The safety population included all randomized patients who consumed at least one dose of study drug and were analyzed by the actual treatment received. Adverse events (AEs) were graded by the investigator according to the Division of AIDS toxicity table (1992) and coded by an adverse event dictionary (MedDRA) [9]. Grade 2–4 AEs (moderate to severe in intensity), treatment-related AEs, and serious AEs were collected. No statistical inferences were performed for these summaries. Changes from baseline in fasting insulin levels, HOMA-IR, QUICKI, and lipid profiles (total cholesterol, high-density lipoprotein, low-density lipoprotein, and triglycerides) were tabulated.

Adherence was calculated as the ratio of the total number of doses taken to the total number of doses that should have been taken. The numerator of the ratio was calculated as the number of tablets dispensed minus the number of tablets returned.

Genotypic summaries were based on differences in the plasma virus sequence from the molecular wild-type strain NL4-3. Reverse transcriptase (RT) and protease (PRO) mutations associated with the development of resistance to antiretroviral therapy as listed by the International AIDS Society (IAS) Drug Resistance Mutation Group were used in the analyses [10].

All analyses were performed using SAS^® ^v8 (SAS Institute, Cary, NC) on a system of UNIX computers.

## Results

### Study Population

Two hundred and seventy-nine (279) subjects were enrolled between May 2004 and March 2005; and the ITT(E) population consisted of 278 subjects. Table 2 summarizes the demographics and baseline characteristics, which were well-matched between the treatment groups. Overall, the study population was predominantly male (79%) and racially diverse (>50% non-white race or ethnicity). Most subjects (82%) had an HIV-1 RNA <100,000 c/mL at baseline. Seven subjects (5%) in the ABC/3TC/ZDV group and 9 subjects (6%) in the ATV+3TC/ZDV group were included in the switch population for suspected ABC hypersensitivity and ATV treatment-limiting toxicities, respectively. No pregnancies or deaths occurred during the study.

**Table 2:** Baseline Demographics and Characteristics

	**ABC/3TC/ZDV (N = 139)**	**ATV+3TC/ZDV (N = 140)**
Median Age, years (range)	38 (19–65)	36 (18–68)
Female Gender, n (%)	30 (22%)	28 (20%)
Race, n (%)		
White	65 (47%)	58 (41%)
Black	44 (32%)	49 (35%)
American Hispanic	26 (19%)	29 (21%)
Other	4 (3%)	4 (3%)
CDC Class C, n (%)	6 (4%)	5 (4%)
Median HIV-1 RNA, log_10 _copies/mL (range)	4.48 (2.3–5.5)	4.64 (2.6–5.6)
HIV-1 RNA ≥ 100,000 copies/mL, n (%)	24 (17%)	25 (18%)
Median CD4^+ ^cell count, (cells/mm^3^) (range)	274 (103–889)	262 (50–749)
CD4^+ ^cell count ≥ 200 cells/mm^3^, n (%)	105 (76%)	97 (69%)
Hepatitis B positive, n (%)	2 (1%)	6 (4%)
Hepatitis C positive, n (%)	9 (6%)	10 (7%)
Hepatitis & B positive, n (%)	0	1 (<1%)

Abbreviations: abacavir sulfate (ABC), atazanavir (ATV), lamivudine (3TC), zidovudine (ZDV)

**Figure 1:**
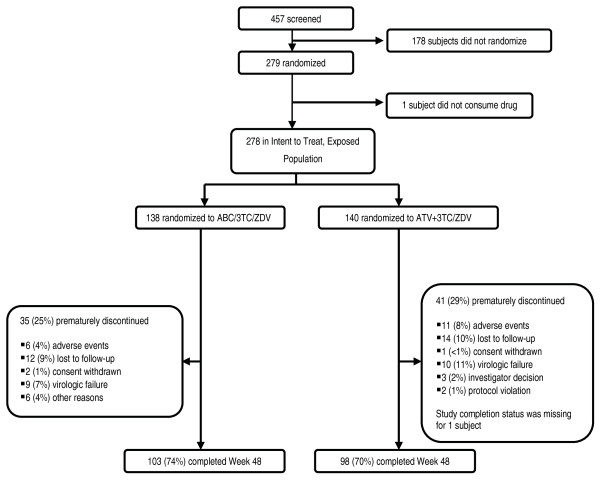
**Subject Disposition**.

Subject disposition through 48 weeks is shown in Figure 1. Data from 3 subjects in the ABC/3TC/ZDV group were not retrievable due to Hurricane Katrina damage in August 2005. In addition, subjects that met the protocol-defined definition of virologic failure were permitted to remain on study if their VL remained less than 1265 c/mL (1/2 log above 400 c/mL). Thus, some subjects appear twice in the summary of study outcome (Table 3), as virologic failures and as study completers. Overall, 74% of subjects in the ABC/3TC/ZDV group and 70% of subjects in ATV+3TC/ZDV group completed the study.

**Table 3:** Study Outcomes at Week 48

	**ABC/3TC/ZDV (N = 138)**	**ATV+3TC/ZDV (N = 140)**
**Completed study**	103 (74%)	98 (70%)
		
**Prematurely withdrawn^1^**	36 (26%)	41 (29%)
		
**Virologic Failures**	18 (13%)	17 (12%)
		
**Primary Reason for Withdrawal^2^, n (%)**		
Adverse event	6 (4%)	11 (8%)
Lost to follow-up	12 (9%)	14 (10%)
Protocol defined virologic failure^3^	16 (12%)	16 (11%)
Subject decision	3 (2%)	1 (<1%)
Protocol violation	0	2 (1%)
Investigator decision	0	3 (2%)
Other^4^	6 (4%)	0

1. One subject in the ATV+3TC/ZDV group had a missing completion status.

2. As reported by study investigators on Study Conclusion case report form.

3. Subjects with confirmed virologic failure with subsequent HIV-1 RNA < 1265 copies/mL were allowed to remain in the study on randomized treatment; therefore the actual number of virologic failures is greater than the number that withdrew from study. In the ABC/3TC/ZDV group, 7 virologic failures completed the study and 2 were lost to follow-up. In the ATV+3TC/ZDV group, 6 virologic failures completed the study and 1 was withdrawn with a protocol violation.

4. Other included (number of subjects): incarceration (2), site not operational due to Hurricane Katrina (3), moved out of state (1).

### Efficacy

**Figure 2:**
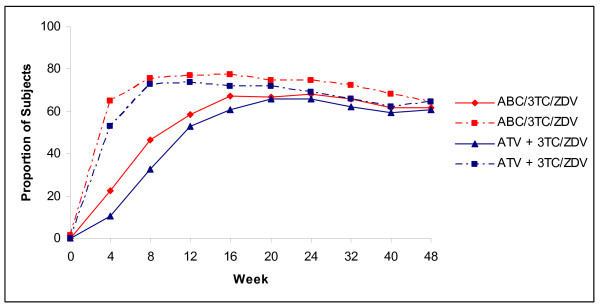
**Proportion of subjects with HIV-1 RNA <50 and <400 copies/mL through Week 48**. Proportion of patients with HIV-1 RNA < 50 copies/mL (solid line) and < 400 copies/mL (dashed line): ITT(E), M/S = F analysis.

The non-inferiority of ABC/3TC/ZDV to ATV+3TC/ZDV was established since 62% (85/138) vs. 59% (83/140) of subjects achieved an HIV-1 RNA <50 c/mL and were considered virologic responders in the primary efficacy analysis at week 48, [ITT(E), M/S = F, 95% CI: -5.9, 10.4] (Figure 2). Similarly, in the Observed analysis, 76% (85/112) vs. 74% (83/112) of subjects in the ABC/3TC/ZDV group vs. ATV+3TC/ZDV group achieved a VL<50 c/mL at week 48, [ITT(E), S = F 95% CI: -6.7, 9.4].

**Figure 3:**
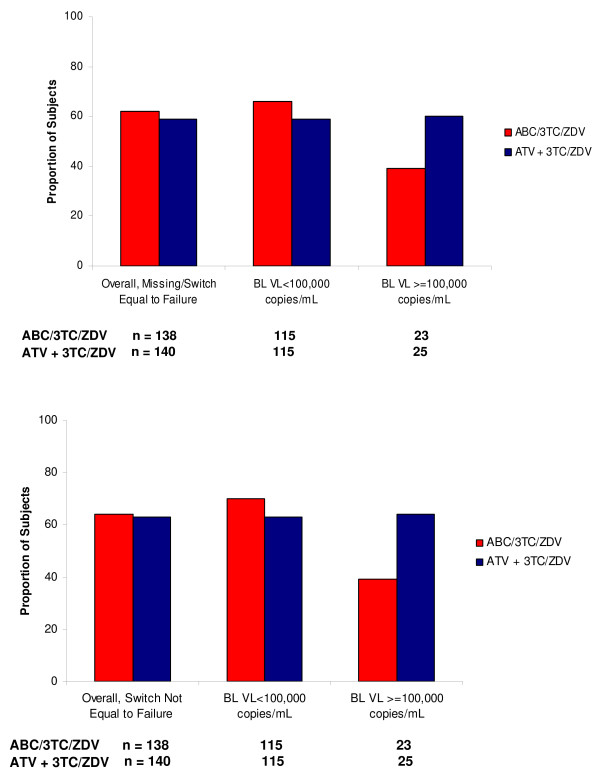
**Proportion of subjects with HIV-1 RNA <50 copies/mL by baseline HIV-1 RNA at Week 48**. Analyses include switch equal to failure (top) and switch not equal to failure (bottom).

In exploratory stratified analyses, similar results were observed in the 230 subjects with baseline VL<100,000 c/mL (ABC/3TC/ZDV vs. ATV+3TC/ZDV), 66% (76/115) vs. 59% (68/115); 95% CI: -5.6, 19.5, suggesting that ABC/3TC/ZDV was virologically non-inferior to ATV+3TC/ZDV in the low viral load stratum (Figure 3). In contrast, among the 48 subjects with baseline VL ≥ 100,000 c/mL (ABC/3TC/ZDV vs. ATV+3TC/ZDV), 39% (9/23) vs. 60% (15/25) achieved a VL<50 c/mL at week 48; 95% CI: -49.2, 7.4, suggesting that ABC/3TC/ZDV did not meet the non-inferiority criterion compared to ATV+3TC/ZDV in the high viral load stratum. Further, the confidence interval suggests that ATV+3TC/ZDV was non-inferior to ABC/3TC/ZDV in the high viral load stratum of this study. Comparable results were seen in stratified Observed analyses at week 48 (ABC/3TC/ZDV vs. ATV+3TC/ZDV); <100,000 c/mL stratum (81% vs. 76%; 95% CI: -7.5, 16.4) and ≥ 100,000 c/mL stratum (50% vs. 65%; 95% CI: -46.2, 15.8). Sensitivity analyses demonstrated no differences in efficacy when subjects with missing data resulting from Hurricane Katrina were excluded.

Secondary analyses of the primary efficacy endpoint in which subjects with toxicity switches were not considered failures, for both the overall population and by viral load strata, resulted in consistent results for both the M = F and observed examinations. At week 48, 67% (93/138) vs. 68% (95/140) of subjects in the ABC/3TC/ZDV vs. ATV+3TC/ZDV group achieved a VL < 400 c/mL [ITT(E), M = F] (Figure 2). Similar results were seen in the Observed analysis; 92% (93/101) vs. 96% (95/99) of subjects in ABC/3TC/ZDV vs. ATV+3TC/ZDV group, respectively. The proportion of subjects (ABC/3TC/ZDV vs. ATV+3TC/ZDV) that achieved a VL < 50 c/mL when toxicity switches were not considered failures was 64% (89/138) vs. 63% (88/140) [ITT(E), M = F] and 80% in each treatment group [(89/111) vs. (88/110)] in Observed analyses, respectively (Figure 3).

Immunologic recovery was observed in both treatment groups at week 48. The median change from baseline in CD4+ cell count was an increase of 147 cells/mm^3 ^in both groups resulting in a median CD4+ cell count of 434 cells/mm^3 ^in the ABC/3TC/ZDV group and 419 cells/mm^3 ^in the ATV+3TC/ZDV group.

**Figure 4:**
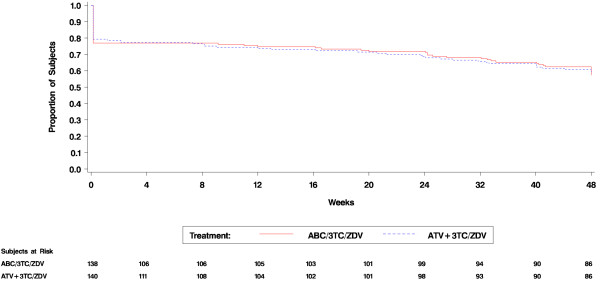
**Time to loss of virologic response (<50 copies/mL)**.

Protocol-defined virologic failure occurred in 18 (13%) vs. 17 (12%) of subjects in the ABC/3TC/ZDV vs. ATV+3TC/ZDV group, respectively (Table 4). Three subjects in the ABC/3TC/ZDV group and 6 subjects in the ATV+3TC/ZDV group met multiple virologic failure criteria. Overall, subjects were more likely to experience virologic rebound after week 24 in the ATV+3TC/ZDV group while more subjects in the ABC/3TC/ZDV group had a VL > 400 c/mL without confirmation at week 48. Regardless of strata, a similar proportion of subjects experienced protocol-defined virologic failure independent of treatment arm. As noted in Figure 4, no difference in time to loss of virologic response between treatment groups was observed. Additionally, adherence to randomized therapy was high (median >97.5%) in both groups.

**Table 4:** Summary of Protocol-Defined Virologic Failure and Drug Resistance at Week 48

	**ABC/3TC/ZDV ** **(N = 138)**	**ATV+3TC/ZDV ** **(N = 140)**
**Virologic Responders**	120 (87%)	123 (88%)

**Protocol-Defined Virologic Failures**	18 (13%)	17 (12%)

1. Less than 1 log_10 _reduction in baseline plasma HIV-1 RNA by week 12	3	3
2. Reduction of plasma HIV-1 RNA < 50 copies/mL on two occasions with a subsequent increase to ≥ 400 copies/mL on two consecutive occasions prior to week 24	2	2
3. Failure to achieve plasma HIV-1 RNA < 400 copies/mL by Week 24	3	4
4. Plasma HIV-1 RNA ≥ 400 copies/mL on consecutive occasions after Week 24	6	11
5. Plasma HIV-1 RNA ≥ 400 copies/mL at Week 48 without confirmation	7	3

**Subjects meeting multiple virologic failure criteria^1^**	3	5

**Post-Baseline Genotype and Phenotype**	17	16

**Subjects with treatment-emergent mutations**	10	11
**Any NRTI-associated Mutations^2^**	8	9
(M184V, M184M/V, L74V, L74I/V D67N, L210W)		
**Any PI-associated mutations**	7	6
(G16E, K20M, L24I, L33V, I54I/L, I62V, V77I, I85I/V, I93I/L)		
**Any NNRTI-associated mutations**	0	1
(K103N)		

Note: Genotype at screening was not performed.

1. In the ABC/3TC/ZDV group, 1 subject met both criteria 2 and 5 and 2 subjects met both criteria 4 and 5; In the ATV+3TC/ZDV group, 1 subject met criteria 2 and 4, 3 subjects met criteria 3 and 4, and 1 subject met criteria 4 and 5.

2. Treatment-emergent mutations that were considered non-significant that also developed included: F77L, I13V, L63L/P, L63P, and E35G.

### Safety

The safety population consisted of 278 subjects (138 ABC/3TC/ZDV; 140 ATV+3TC/ZDV) who were analyzed based on treatment received. One subject randomized to ABC/3TC/ZDV was dispensed ATV+3TC/ZDV for the duration of the study and was analyzed based on the actual treatment received. The overall incidence of Grade 2–4 adverse events was similar between arms; 72% in each group. Treatment-related Grade 2–4 adverse events occurred less frequently in the ABC/3TC/ZDV group with notably different toxicities between groups (Table 5). Hyperbilirubinemia was the most frequently reported adverse event occurring in 21% of subjects in the ATV+3TC/ZDV group followed by nausea in 11% of subjects in the ABC/3TC/ZDV group and 4% of subjects in the ATV+3TC/ZDV group. Treatment-related serious adverse events were reported by 10 subjects (7 ABC/3TC/ZDV; 3 ATV+3TC/ZDV). Suspected abacavir hypersensitivity reactions (ABC HSR) were considered serious adverse events and were reported in 7 (5%) subjects receiving ABC/3TC/ZDV of which 6 were classified as Grade-2 and 1 as a Grade-1 event; none of the subjects experiencing a suspected ABC HSR discontinued study. In the ATV+3TC/ZDV group, 3 (2%) subjects reported anemia (2-Grade 3, 1-Grade 4) each requiring blood transfusions, two of whom discontinued from study.

**Table 5:** Grade 2–4 Treatment-Related Adverse Events & Laboratory Abnormalities Occurring in ≥ 2% of Subjects

	**ABC/3TC/ZDV (N = 138)**	**ATV+3TC/ZDV (N = 140)**
**Any Grade 2–4 event (Grade 3–4)**	**30% (7%)**	**47% (25%)**
Hyperbilirubinemia	0	21% (15%)
Nausea	11% (<1%)	4% (0)
Headache	4% (<1%)	4% (0)
Neutropenia	4% (4%)	5% (4%)
Fatigue	5% (<1%)	2% (0)
Suspected Abacavir Hypersensitivity^1^	5% (0)	0
Vomiting	2% (0)	2% (0)
Anemia	<1% (<1%)	3% (3%)
Ocular Icterus	0	4% (2%)
Abdominal pain	2% (0)	<1% (0)
Increased Creatinine Phosphokinase	0	2% (1%)
Rash	0	2% (0)

^1 ^Includes 1 report of a Grade-1 suspected abacavir hypersensitivity reaction

Treatment-emergent Grade 3–4 laboratory abnormalities were infrequent and generally comparable between groups (Table 5). Grade 3–4 hyperbilirubinemia occurred in 15% of subjects treated with ATV+3TC/ZDV. Two subjects discontinued study for atazanavir-related treatment toxicities, 1 for jaundice and 1 for hyperbilirubinemia. Additionally, 4 subjects receiving atazanavir elected to switch to fosamprenavir for asymptomatic, grade 3 hyperbilirubinemia without clinical evidence of jaundice or scleral icterus. Grade 3–4 neutropenia was reported in 10 subjects; 4% ABC/3TC/ZDV; 4% ATV+3TC/ZDV. Changes in fasting lipids and glucose were comparable in both treatment groups (Table 6). At week 48, median triglycerides were 126 mg/dL in the ABC/3TC/ZDV group compared with 110 mg/dL in the ATV+3TC/ZDV group. However, all median fasting lipid parameters were below NCEP ATP III thresholds for both groups at week 48 [11,12]. Changes in glucose, insulin sensitivity and insulin resistance were minimal and comparable between groups. The use of concurrent lipid lowering medications was minimal; 5 subjects received statins (2 ABC/3TC/ZDV; 3 ATV+3TC/ZDV) and 1 subject in the ABC/3TC/ZDV group received a fibrate.

**Table 6:** Median Fasting Lipid and Metabolic Parameters at Baseline and Week 48

	**ABC/3TC/ZDV** **N = 138**			**ATV+3TC/ZDV** **N = 140**		
Median (range)	Number Tested (Baseline, Week 48)	Baseline	Week 48	Number Tested (Baseline, Week 48)	Baseline	Week 48

TC (mg/dL)	131, 93	162 (95–255)	176 (102–305)	137, 93	160 (71–276)	171 (68–288)

LDL (mg/dL)	127, 89	98 (6–165)	99 (20–198)	133, 92	97 (31–200)	101.5 (10–200)

HDL (mg/dL)	131, 93	36.5 (6–74)	44 (21–75)	135, 93	33 (7–77)	44 (18–90)

TG (mg/dL)	131, 93	113 (38–621)	126 (42–1231)	137, 93	117 (41–559)	110 (44–407)

Glucose (mg/dL)	119, 91	95 (60–199)	98 (5–268)	126, 93	89.5 (58–131)	95 (72–166)

Insulin (μIU/mL)	117, 79	10 (2–128)	13 (2–56)	119, 83	10 (2–61)	12 (4–132)

HOMA-IR^+^	110, 83	2.2 (0.4–37.5)	2.9 (0.4–17.3)	112, 87	2.4 (0.4–19.5)	3 (0.9–51.6)

QUICKI^++^	110, 83	0.59 (0.34–1.05)	0.54 (0.38–1.00)	112, 87	0.58 (0.37–1.00)	0.54 (0.32–0.77)

^+^HOMA-IR = [Fasting Insulin (μIU/mL) × Fasting Glucose (mg/dL))/(22.5 × 18)];

^++^QUICKI = 1/[log Fasting Insulin (μIU/mL)] + [log Fasting Glucose (mg/dL)]

There were no myocardial infarctions reported in the ABC/3TC/ZDV arm. Two cases of non-cardiac chest pain (both confirmed by a negative diagnostic evaluation) were reported in the ABC/3TC/ZDV arm. In the ATV + 3TC/ZDV arm, 1 case of confirmed myocardial infarction/coronary occlusion was reported. All 3 cases were reported as not-related to study drug.

### Viral Resistance

Thirty-five subjects (13%) [18 ABC/3TC/ZDV; 17 ATV+3TC/ZDV] met one or more protocol-defined virologic failure criteria. Paired genotypes and phenotypes were available for 17/18 subjects in the ABC/3TC/ZDV group and 16/17 subjects in the ATV+3TC/ZDV group at baseline and at the time of virologic failure (Table 4).

Among the 35 subjects with virologic failure, 11.4% had primary NRTI or NNRTI mutations and 5.7% had primary PI-associated resistance mutations at baseline. Three subjects (2 ABC/3TC/ZDV and 1 ATV+3TC/ZDV) with virologic failure had primary ABC- and/or 3TC-related mutations at baseline. Primary PI mutations were present at baseline in 2 subjects experiencing virologic failure [1 D30N (ABC/3TC/ZDV); 1 L90M (ATV+3TC/ZDV)] and the NNRTI-associated K103N mutation was present in 1 subject receiving ATV+3TC/ZDV. Treatment-emergent ABC- and/or 3TC-associated mutations occurred in 10 subjects failing ABC/3TC/ZDV, the majority of which developed the M184V mutation and to a lesser extent, minor PI mutations suggesting these subjects had virus harboring PI drug-resistance at baseline. In the ATV+3TC/ZDV group, 11 subjects had treatment-emergent mutations; 11 subjects developed the M184V nucleoside mutation, 1 subject developed K103N, and 6 subjects developed minor PI mutations. No major ATV-related mutations developed over 48 weeks.

Treatment-emergent drug resistance occurred in subjects experiencing virologic failure in both treatment arms. Among the 35 subjects (13%) experiencing virologic failure, the M184V mutation developed most commonly in 46% (16/35) of subjects. Among subjects with paired phenotypic data, 45% (15/33) had no drug-associated reduced susceptibility present while 55% (18/33) had reduced phenotypic susceptibility present on treatment. No subject had primary PI resistance at the time of virologic failure as defined by IAS-USA guidelines [10]. These results suggest that subjects failing on either treatment regimen may have therapy options available given that cross-resistance to other members in the nucleoside and protease inhibitor classes was not seen.

## Discussion

ACTION was one of the first studies to evaluate the safety and efficacy of ABC/3TC/ZDV compared to atazanavir, the latter which was approved approximately 1 year prior to initiation of present study. This study demonstrated the virologic non-inferiority of ABC/3TC/ZDV compared to ATV+3TC/ZDV in this study population over 48 weeks. Virologic and immunologic responses were similar between the treatment arms (ABC/3TC/ZDV vs. ATV+3TC/ZDV) given 62% vs. 59% of subjects achieved an HIV-1 RNA <50 c/mL and the median CD4+ cell count increase was 147 cells/mm^3 ^in both groups at week 48. Similar responses were also evident in subjects with a lower baseline viral load, as 66% and 59% of subjects in the ABC/3TC/ZDV group and ATV+3TC/ZDV group achieved the primary endpoint. However, virologic response was suboptimal in the ABC/3TC/ZDV (39%) group of subjects with a higher baseline viral load (≥ 100,000 c/mL) compared to those in the ATV+3TC/ZDV group (60%). The small number of subjects in the higher viral load strata did not account for the large difference between treatment arms since the study was stratified *a priori*. However, atazanavir in combination with lamivudine/zidovudine performed well regardless of baseline viral load.

The rate of virologic failure was somewhat higher than expected in this study in both treatment arms, likely due to the use of multiple criteria for virologic failure, (13% ABC/3TC/ZDV; 12% ATV+3TC/ZDV). A conservative definition of virologic failure was adopted partly in response to the results of the ACTG 5095 study and subsequent change in the Department of Health and Human Services guidelines for antiretroviral therapy reserving triple nucleoside regimens as alternative first line options. Differential treatment responses with ABC/3TC/ZDV in patients with higher baseline viral loads have been observed rarely; reduced responses were first observed in those with higher baseline VL (>100,000 c/mL) when compared to a PI-based regimen in Study CNA3005, and more recently in the ACTG 5095 study which demonstrated an inferior virologic response of ABC/3TC/ZDV compared to pooled EFV-containing arms with dual and triple nucleoside backbones regardless of pre-treatment VL (< or ≥ 100,000 c/mL) [2,5].

Since no one particular virologic failure criteria was met more often than another and reported medication adherence was high, this suggests that subjects receiving ABC/3TC/ZDV are not more likely to experience early virologic non-response than virologic response followed by viral rebound. For select subjects with baseline VL < 100,000 c/mL, this study demonstrated that subjects receiving ABC/3TC/ZDV have an equal opportunity for achieving virologic success as those receiving an ATV-containing regimen without low-dose ritonavir boosting.

The unexpected appearance of treatment-emergent PI mutations among subjects receiving ABC/3TC/ZDV may have been attributed to the emergence of virus harboring low levels of PI mutations undetectable at baseline from either prior PI experience or acquisition of drug-resistance virus. Similarly, the development of the K103N mutation in a subject receiving ATV+3TC/ZDV suggests virus with prior exposure to NNRTIs. Phenotypic resistance toward either treatment regimen was observed infrequently suggesting that subjects failing either treatment may have within-class therapy options available given that cross-resistance to other members in the nucleoside and protease inhibitor classes was not seen.

Both regimens were generally well-tolerated and a comparable number of subjects, 4% vs. 8%, discontinued study resulting from adverse effects in the ABC/3TC/ZDV vs. ATV+3TC/ZDV group, respectively. Investigators were permitted to switch patients off atazanavir for the management of asymptomatic hyperbilirubinemia as per practice in previous initial studies conducted by BMS [13].

Changes in fasting lipids and metabolic parameters were minimal in this study. Median triglycerides were somewhat higher in the ABC/3TC/ZDV group compared to the ATV+3TC/ZDV group (126 vs. 110 mg/dL); increases in total cholesterol (176 vs. 171 mg/dL) were driven by favorable increases in HDL cholesterol, however, all other lipid parameters were essentially unchanged from baseline. The minimal changes in fasting lipids are of limited clinical significance given all parameters were below target NCEP ATP III recommendations and were similar to other published reports [8,14-16]. No differences in serum fasting glucose, insulin, HOMA-IR or QUICKI were observed between treatment arms over 48 weeks.

A fixed dose combination of ABC/3TC/ZDV provides an effective, generally well-tolerated, and simplified triple nucleoside regimen for select antiretroviral naïve subjects with baseline viral loads < 100,000 c/mL. This dosing regimen may be advantageous as an alternative regimen for subjects with pre-existing co-morbidities, history of polypharmacy with drug-drug interaction potential for whom an NNRTI or PI-containing regimen would be challenging. For select patients who cannot tolerate the recommended first line therapy options, both ABC/3TC/ZDV and ATV+3TC/ZDV have demonstrated both efficacy and safety in this study conferring an important role for these therapies in both newly HIV-1 infected adults as well as in the aging HIV-1 infected population of the United States.

## Conclusion

A fixed dose combination of ABC/3TC/ZDV provides an effective, generally well-tolerated, and simplified triple nucleoside regimen for select antiretroviral naïve subjects with baseline viral loads < 100,000 c/mL. This dosing regimen may be advantageous as an alternative regimen for subjects with pre-existing co-morbidities, history of polypharmacy with drug-drug interaction potential for whom an NNRTI or PI-containing regimen would be challenging. For select patients who cannot tolerate the recommended first line therapy options, both ABC/3TC/ZDV and ATV+3TC/ZDV have demonstrated both efficacy and safety in this study conferring an important role for these therapies in both newly HIV-1 infected adults as well as in the aging HIV-1 infected population of the United States.

## Competing interests

PK has participated in speakers bureaus and received research grants from GlaxoSmithKline and Merck. PS and ALM have no relevant competing interests. ED has research grants from Roche Laboratories, Inc. and Gilead Sciences, Inc. and is a Consultant to and/or Advisory Board Member of Bristol-Myers Squibb Company, Gilead Sciences, Inc. (Regional Consultant), GlaxoSmithKline, Roche Laboratories, Inc., and Vertex Pharmaceuticals. PP, AF, and MS are employed by GlaxoSmithKline. DMC was former employee of GlaxoSmithKline.

## Authors' contributions

PNK and PP participated in the analysis and interpretation of the data and the development of the manuscript. AF, DM, and MS participated in the conception and design of the study, analysis, and interpretation of the data, and development of the manuscript. PS, AL, ED participated in the analysis of the data and development of the manuscript. All authors read and approved the final manuscript.
